# Association between household crowding at birth and mental health outcomes in school-aged children: retrospective analysis of data from the Born in Bradford cohort, UK

**DOI:** 10.1136/bmjph-2026-005483

**Published:** 2026-10-29

**Authors:** Joanna Klaptocz, Helen Abigail Blake, Kimon Krenz, Cathy Knamiller, Rosemary RC McEachan, Marcella Ucci, Jessica Sheringham

**Affiliations:** 1 UCL Research Department of Primary Care and Population Health, University College London, London, UK; 2 UCL Bartlett Faculty of the Built Environment, University College London, London, UK; 3 Population Health Improvement (PHI-UK), Bradford, UK; 4 Bradford Institute for Health Research, Bradford Teaching Hospitals NHS Foundation Trust, Bradford, UK

**Keywords:** Mental Health, Epidemiology, Public Health

## Abstract

**Introduction:**

Previous research suggests household crowding is associated with poorer child mental health, but findings are inconsistent. Longitudinal data is required, especially from deprived settings where crowding is most prevalent and in ethnically diverse UK settings where living in larger family units is more commonplace. It is uncertain whether features of nearby green space may modify associations between crowding and child mental health.

**Methods:**

We analysed data from 3543 children in the Born in Bradford study, a longitudinal cohort representing residents of the ethnically diverse and relatively deprived city of Bradford, UK. We used ordinal logistic regression to investigate the association between exposure to household crowding (self-reported people per bedroom) at birth and child mental health score (normal, borderline, raised) 7–14 years later according to parent-reported Strengths and Difficulties Questionnaire (SDQ), adjusting for gender, ethnicity and socio-economic position. We explored effect modification by ethnicity and green space measures.

**Results:**

Greater levels of household crowding at birth were not associated with being in a higher SDQ category 7–14 years later; in crude and adjusted analyses, the fully adjusted OR was 0.89, 95% CI 0.76 to 1.05. No effect modification by the main ethnic groups or parental satisfaction with surrounding green space at the time of outcome measurement was observed.

**Conclusion:**

The relationship between household crowding and child mental health is complex. In this study of an ethnically diverse population, where crowding was relatively prevalent, no relationship was found. Household composition, age, housing condition and family and community support likely play important roles.

**WHAT IS ALREADY KNOWN**

- There is inconsistent evidence for an association between household crowding and child mental health. There is a need for evidence from longitudinal studies in under-represented populations including ethnic minorities and deprived communities and for exploration of the role of protective factors like green space.

**WHAT THIS STUDY ADDS**

- This cohort analysis finds no association between objective measures of household crowding at birth and child mental health outcomes between the ages of 7 and 14, nor evidence for effect modification by ethnicity or green space measures.

**HOW THIS STUDY MIGHT BENEFIT RESEARCH, PRACTICE OR POLICY**

- Household crowding may not always be associated with poorer mental health outcomes in children. Given contradictory findings, exploration of individual and community level protective factors including family support, age-specific effects and use of subjective and objective measures for defining household crowding and protective factors like green space is required.

## Introduction

 The home and its surrounding environment are key social determinants of health and well-being.^1^ Household crowding, meaning the space available to individuals within a home, is an important element of the home environment linked to health and well-being.^2^ Levels of household crowding in urban areas of the UK and Europe are rising.^3,4^

A systematic review of household crowding highlighted a paucity of evidence for an association with mental health, especially among children.^2^ Children are particularly susceptible to environmental factors^5^ and mental health in childhood is an important predictor of adult mental health.^6^ Qualitative work in the UK identified household crowding as a pathway through which home environment affects family psychological well-being.^7,8^ Quantitative studies in the USA^9^ and the UK^10^ suggest that high levels of crowding are associated with poorer child mental health outcomes. Despite this, effect sizes and strengths of associations vary considerably. UK evidence in deprived settings where socioeconomic conditions increase the likelihood of living in crowded conditions is scarce. Ethnically diverse communities where families may live in larger households, are also underrepresented. It is not known whether vulnerability to the effects of household crowding differs across ethnic groups.^3^ A recent review on housing exposures and child mental health calls for use of standardised, objective measures and cohort designs to identify critical periods of exposure.^11^

Finding solutions to the problem of household crowding is challenging. There are not enough sufficiently sized, affordable, urban homes for families,^12,13^ relocation to other areas may not be favourable or feasible.^12^ High-quality green spaces where children can play have been linked to better mental health outcomes in children^14^ and might help mitigate negative effects of household crowding.

Given these evidence gaps, this paper addresses the following questions:

Is household crowding exposure at birth associated with poorer child mental health outcomes at school age in a deprived and ethnically diverse urban population in the UK?

Does the quality of, access to, and use of green space surrounding the home modify this association?

Does the association between household crowding and child mental health vary between ethnic groups?

## Methods

### Study design and participants

This study used data from the Born in Bradford longitudinal cohort study. 12 453 mothers were recruited during pregnancy at Bradford’s main maternity unit between 2007 and 2011. The data used in this study were extracted from anonymised responses to questionnaires at baseline, during late pregnancy and follow-up assessments between 2017 and 2021 when children were 7–14 years old. Full methods were previously published.^15^ The study protocol was pre-registered on Open Science Framework.^16^

### Data

Measures of household crowding commonly used in research include people per bedroom, people per room and square metres per person.^10^ Accepted thresholds for ‘overcrowding’ are: more than two people per bedroom, more than one person per room and less than 15.3 square metres per person.^10^

People per bedroom was used as the primary measure due to its relative completeness and closer relation to the bedroom standard, widely used in England including in local housing allocation policy.^17^ To avoid information loss, a continuous measure of people per bedroom was calculated at baseline by dividing self-reported number of household members by self-reported bedrooms in the home, including bedsitting and spare rooms.

Alternative measures created for sensitivity analysis were as follows:

People per bedroom was regrouped into a dichotomous variable using the overcrowding threshold of more than two people per bedroom^10,18^ and into data-defined tertiles. Changing household crowding was captured by subtracting people per bedroom at the study end from the study start. Cumulative overcrowding was estimated from those meeting the overcrowding threshold at both the start and end; either the start or end but not both; and at neither the start nor end.

People per room and square metres per person were calculated using baseline self-reported number of household members and number of rooms and square metres of the property lived in, based on surveyors’ reports from Energy Performance Certificates (EPC). EPC inspection dates ranged from 2008 to 2021 and may not have accurately reflected property characteristics at baseline.

### Primary outcome

Child mental well-being was measured using the parent-completed standardised Strengths and Difficulties Questionnaire (SDQ),^19^ collected when children were aged 7–14 years (study end). A total difficulties score was calculated from four domains: emotional symptoms, conduct, hyperactivity/inattention and peer relationships. The score, ranging from 0 to 40, was categorised using standard cut-offs,^20^ into ‘normal’ (0–13 points), ‘borderline’ (14–16 points) and ‘raised’ (17–40 points).

### Covariates

Covariates that might confound the association between household crowding and child mental health were identified from a literature search, the Born in Bradford Data dictionary^21^ and discussion among team members with housing and health expertise. A Directed Acyclic Graph (DAG) (appendix figure 1) illustrates assumptions made. Online appendix table 1 illustrates the classification, type and timing of covariates and describes the nature of assumed relationships depicted in the DAG.

Mother’s age and child’s sex were collected at study registration (baseline), child’s age at SDQ completion. Age was treated as a continuous variable for all main analyses and grouped into three categories (below 9, 9–11 and over 11) for stratified analysis.

Ethnicity was self-reported in 16 categories.^22^ We regrouped these into three broader groups: Asian, White and Other ethnic groups. In addition to being treated as a confounder, effect modification by ethnicity was also explored.

Indicators of individual and area-level deprivation included: self-reported financial status, maternal education and Index of Multiple Deprivation 2010 (IMD). Self-reported financial status was explored using the question: “How well would you say you or you and your husband/partner are managing financially these days?” Response options ‘Just getting by’, ‘quite difficult’ and ‘very difficult’ were coded as ‘struggling’; ‘living comfortably’ and ‘doing alright’ were coded as ‘not struggling’. Maternal education was categorised as having qualifications of, or equivalent to, below 5 GCSE level, at 5 GCSE level, A-Level, above A-Level and other. Household postcodes were linked to 2010 IMD score,^23^ an area-level ranking incorporating seven domains (income, employment, education, health, crime, barriers to housing and services, and living environment). Due to relatively high levels of deprivation in Bradford, IMD scores were grouped into local, not National, quintiles. 1 represented the most deprived and 5 represented the least deprived. IMD categories 4 and 5 had lower numbers here and were combined.

Housing tenure was self-reported and categorised as owner (mortgage or outright), private rented, council rented or other (including rent free and squatting).

Marital and cohabitation status were self-reported as married and living with partner, not married and living with partner, and not living with partner.

Green space measures were treated as potential effect modifiers of the association between household crowding and child mental health and were measured as follows:

Parent self-rated satisfaction (satisfied, neutral, dissatisfied) with green spaces including public parks, playing fields or other natural habitats (eg, woodland) in the local area, child-reported use of green spaces (study end), grouped into ‘rarely’, ‘some weekdays or some weekends’, ‘some weekdays and some weekends’ and ‘most days’; distance from the child’s address to the closest public park or garden in metres and parent perceived availability of safe outdoor space to play (yes or no) were measured at the study end and treated as proxy measures for green space satisfaction and use at the study start.

Normalised Difference Vegetation Index (NDVI) was measured within a 300 m straight line zone surrounding the child’s home address as a marker of vegetation/‘greenness’.^24^

Despite originally being considered as potential confounders, maternal mental health^10,25,26^ and home moves over the course of the study were, after review of the literature and discussions within the research group, felt to be potential mediators on the causal pathway between crowding and children’s mental health and so were excluded from the final model to avoid overadjustment but included in sensitivity analyses under an alternative assumption that they were confounders.

Current maternal mental health was measured using the standardised adult mental health questionnaire General Health Questionnaire- 28 (GHQ-28).^27^

The number of moves, a marker of housing instability,^28,29^ could also occur due to crowding.

### Analysis

All analyses were conducted in StataBE 17.^30^

Each variable was examined for sparsity, extreme outliers and missing data.

Where possible, variables were kept in original forms, with re-grouping as described above to prevent sparsity.

The IQR method^31^ was used to identify extreme outliers (third quartile+1.5*IQR or first quartile–1.5*IQR). Extreme outliers and impossible values were treated as missing.

Age, gender, ethnicity, self-reported financial status and IMD of participants with any missing observation were compared with those with complete data. Multiple imputation was not performed as no variables met the planned cut-off with >2% missing data.

Mean and SD or median and IQR were examined for continuous parametric and non-parametric variables respectively. P values were calculated using χ^2^ test for comparing proportions or t-test for comparing means as appropriate.

Crude associations between people per bedroom and covariates were examined. As people per bedroom was positively skewed, Spearman, Kruskal-Wallis and Wilcoxon tests were used for continuous, categorical and binary variables respectively. Crude associations between SDQ category and covariates were examined using χ^2^ and ordinal logistic regression.

Starting with a crude ordinal regression model for the association between people per bedroom and SDQ score, covariates, identified by the DAG, were added individually and the change in OR for the association between household crowding and SDQ score was observed. Certain covariates: age, ethnicity, self-reported financial status, maternal education, IMD and a green space measure were chosen to be included in the multivariable model regardless of their effect on the crude OR. A threshold of a ≥5% change in the OR was used to determine whether remaining covariates were included in the multivariable model. This threshold was met by all remaining covariates.

Multivariable ordinal logistic regression adjusted for confounding, by age, sex, ethnicity, self-reported financial status, maternal education, IMD, marital status, tenure, maternal age and satisfaction with green space were added sequentially. Satisfaction with green spaces was the principal green space measure due to relative completeness, producing the ‘best fitting model’ according to Akaike and Bayesian information criteria and capturing green space ‘useability’.

The final multivariable model OR reflected the odds of being in a higher SDQ category for each unit increase of one person per bedroom, holding all other variables constant. Brant testing was used to confirm that the proportional odds assumption was met.

To investigate effect modification by green space and ethnicity, an interaction term with people per bedroom was included in the fully adjusted model. Likelihood ratio test (lrtest) compared models with and without interaction terms. A lrtest p value of<0.05 was interpreted as evidence of effect modification.

### Sensitivity analysis

Logistic regression was conducted using alternative exposure measures: people per room and square metre per person; alternative groupings of the exposure (binary (crowded/not crowded) and tertiles defined by the data (low, medium, high crowding)); change in people per bedroom and cumulative overcrowding.

Effect modification was explored by alternative green space measures.

Logistic regression was conducted including maternal mental health and number of moves under the alternative assumption that they were confounders and not mediators.

Stratified analysis by different age categories was performed to explore the possibility of age specific associations due to the relatively broad age-range of the sample.

A linear regression model was used to explore the association between people per bedroom and SDQ score in its continuous, un-categorised form. Due to non-normal distribution of residuals, this was not used as the primary model in this analysis.

### Patient and public involvement

The Born in Bradford study encourages community involvement in all research stages,^32^ informal discussion with stakeholders identified household crowding as a priority research topic. We consulted a group of 10 Bradford residents regarding results analysis. While not a formal part of data analysis, they informed interpretations in the discussion. Study results were disseminated at a community household crowding event.

## Results

### Participants

Figure 1 depicts how the cohort of 3913 children, of whom 3543 had complete data, was derived from the Born in Bradford cohort.

**Figure 1:**
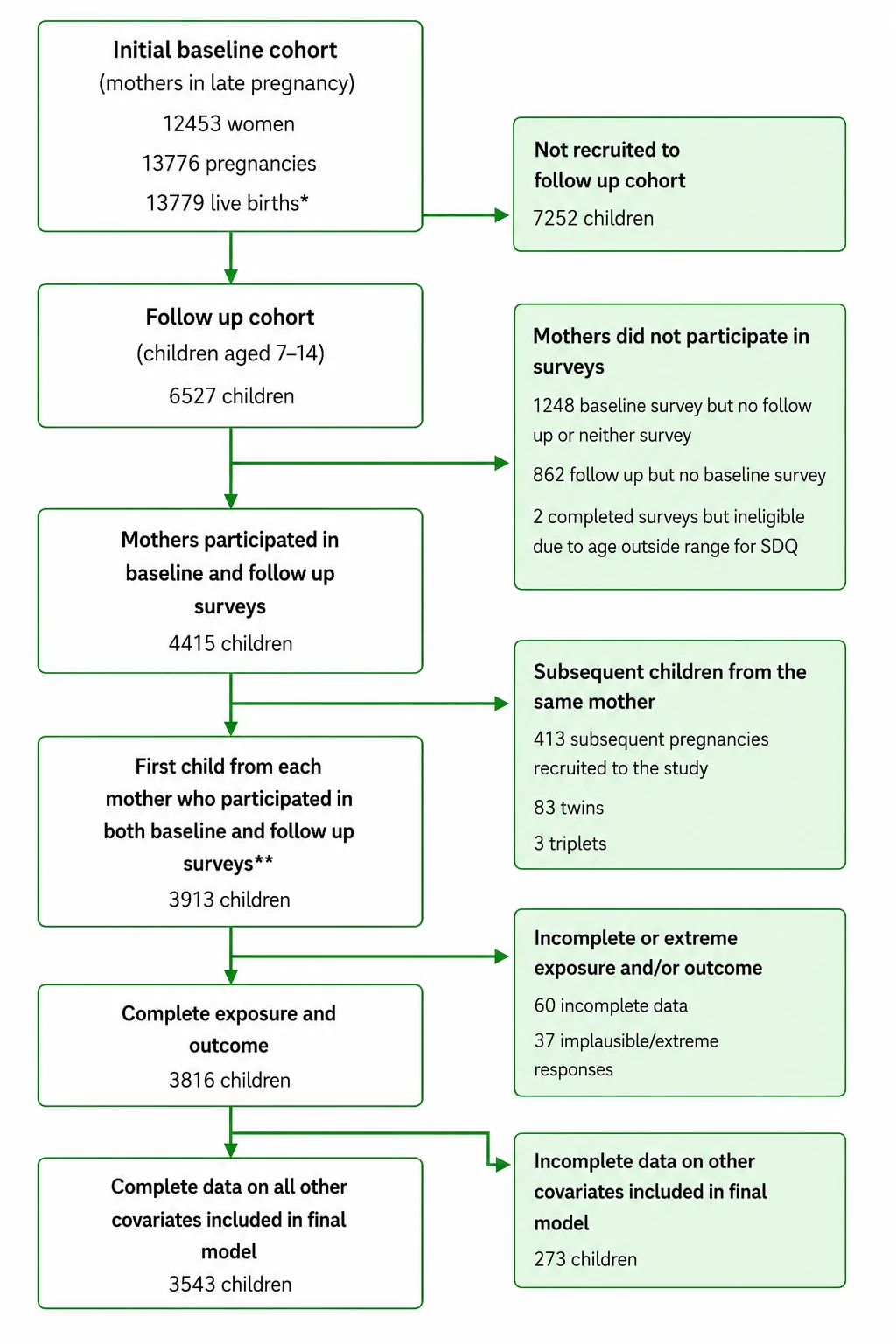
Flowchart for the selection of the final study cohort. *Includes multiple pregnancies (twins, triplets) and repeat pregnancies over the duration of follow up. ** Several mothers had multiple children over the course of the study, once recruited, subsequent children were automatically offered participation in the study. We selected the first child to be enrolled in the study so that results were not biased by the inclusion of multiple, related children growing up in the same household. SDQ, strengths and difficulties questionnaire.

Table 1 depicts characteristics of study participants with respect to variables in main analyses. Online appendix table 2 contains sensitivity analysis variables.

**Table 1:** Characteristics of participants, n=3913

Variable	Number (%), mean (SD) or median† (IQR)	Number of observations		
		Complete	Missing	Extreme or impossible
Crowding and Mental health measures				
People per bedroom, median (IQR)	1.50 (1.3–2.0)	3862	14	37
Strengths and difficulties (SDQ) score		3866	47	0
Normal (0–13 points)	3129 (81)			
Borderline (14–16 points)	290 (8)			
Raised (17–40 points)	447 (12)			
Other covariates				
Age (years) mean (SD, min/max)	9.86 (1.0, 7–14)	3913	0	0
Gender		3913	0	0
Male	2002 (51)			
Female	1911 (49)			
Ethnicity‡		3913	0	0
Asian	2437 (62)			
White	1229 (31)			
Other	247 (6)			
Self-reported financial status		3894	19	0
Not struggling	2696 (69)			
Struggling	1198 (31)			
Index of multiple deprivation 2010 (IMD)		3882	31	0
5 (least deprived)	120 (3)			
4	459 (12)			
3	730 (18)			
2	1074 (28)			
1(most deprived)	1499 (39)			
Marital/cohabitation status		3904	9	0
Married & living with partner	2925 (75)			
Not married & living with partner	488 (12)			
Not living with partner	491 (13)			
Maternal education		3821	92	0
<GCSE	845 (22)			
GCSE equivalent	1139 (30)			
A-level equivalent	571 (15)			
>A-level	1047 (27)			
Other	219 (6)			
Maternal age (years), mean (SD)	27.90 (5.6)	3913	0	0
Tenure		3860	53	0
Owner	2636 (68)			
Private rented	557 (14)			
Council rented	350 (9)			
Other (incl. rent free)	317 (8)			
Satisfaction with green spaces		3813	100	0
Satisfied	2901 (76)			
Neutral	459 (12)			
Dissatisfied	453 (12)			

* All available data is used to here for children from unique families who participated in both baseline and follow-up surveys.

† Median is used in place of mean for variables with a skewed distribution

‡ The Asian category includes Indian, Pakistani, Bangladeshi, Chinese or other Asian backgrounds, of which 84% were Pakistani. Other ethnic group includes all Black, African, Caribbean backgrounds and any other non-White, non-Asian ethnic groups.

IMD, index of multiple deprivation; SDQ, strengths and difficulties questionnaire.

Participants who completed follow-up were more likely than the baseline cohort to be Asian (2437/3913 (62%) vs 2770/6382 (43%)) and live in areas of higher deprivation 1499 (39%) vs (2236 (36%)) but were similar in terms of sex and self-reported financial status (online supplemental table 3). Those with complete and missing data were broadly similar with small differences in ethnicity and age (online appendix table 4).

Table 2 shows people per bedroom was higher in those of Asian ethnicity, struggling financially and living in areas of higher deprivation. SDQ scores were higher in boys and those of white ethnicity, struggling financially and living in areas of higher deprivation. Appendix figure 2 shows the distribution of household crowding in the sample.

**Table 2:** Crowding and SDQ score by age, gender, ethnicity and socio-economic status, n=3543

Variable	People per bedroom (median, IQR)	P value*	SDQ score (number, (%))			P value†	N
			Normal	Borderline	Raised		
Age		0.200				0.221	
<9	1.50 (1.3–2.0)		549 (81)	57 (8)	75 (11)		681
9-11	1.50 (1.3–2.0)		2033 (81)	191 (8)	297 (12)		2521
>11	1.50 (1.3–2.0)		291 (85)	17 (5)	33 (10)		311
Gender		0.910				<0.001	
Male	1.50 (1.3–2.0)		1409 (78)	152 (9)	238 (13)		1799
Female	1.50 (1.3–2.0)		1464 (84)	113 (7)	167 (10)		1744
Ethnicity		<0.001				<0.001	
Asian	1.67 (1.3–2.0)		1849 (84)	166 (8)	192 (9)		2207
White	1.33 (1.3–1.7)		852 (76)	85 (8)	191(17)		1128
Other	1.50 (1.3–2.0)		172 (83)	14 (7)	22 (11)		208
Self-reported financial status		0.003				<0.001	
Not struggling	1.50 (1.3–2.0)		2036 (83)	168 (7)	242 (10)		2446
Struggling	1.71 (1.3–2.0)		837 (76)	97 (9)	163 (15)		1097
IMD		<0.001				0.003	
4/5 (least deprived)	1.33 (1.0–1.7)		448 (84)	29 (5)	58 (11)		535
3	1.50 (1.3–1.8)		567 (84)	34 (5)	73 (11)		674
2	1.67 (1.3–2.0)		783 (79)	97 (10)	106 (11)		986
1 (most deprived)	1.67 (1.3–2.0)		1075 (80)	105 (8)	168 (13)		1348

* Kruskal Wallis test for difference in median between groups

† Chi-squared test for differences between groups

IMD, index of multiple deprivation; IQR, interquartile range; N, total number of observations; SDQ, strengths and difficulties questionnaire.

Associations between household crowding and child mental health

For each unit increase in people per bedroom, there was no increase in the odds of a higher SDQ category in crude or fully adjusted analyses (table 3).

**Table 3:** Association between household crowding and child mental health, n=3543

Variable	Increasing SDQ category, OR (CI)	P value*
Crude		
People per bedroom	0.95 (0.82 to 1.10)	0.474
Fully adjusted		
People per bedroom	0.89 (0.76 to 1.05)	0.178
Alternative measures of household crowding, fully adjusted		
People per room	1.16 (0.91 to 1.49)	0.227
Square metres per person	0.99 (0.98 to 1.00)	0.106

* Two-sided Wald z-tests

CI, confidence interval; OR, odds ratio; SDQ, strengths and difficulties questionnaire.

Sensitivity analyses using alternative household crowding measures, people per room and square metres per person were consistent with this finding (table 3).

Sensitivity analysis including mediators (maternal mental health and number of home moves) as confounders changed the OR estimate no more than 1.5% (online appendix table 5c). Inclusion of extreme outliers produced a <0.5% change in the OR. Grouping measures of household crowding using accepted binary cut-offs and tertiles consistently showed no evidence of association (online supplemental table 5a).

Change in people per bedroom and cumulative overcrowding over time did not show consistent patterns of association with SDQ (online supplemental table 5b).

Stratified analysis by age groups did not find age-specific effects (online supplemental table 5e) and the p value of the lrtest for effect modification by age category was 0.560.

Sensitivity analysis using a linear regression model found a regression coefficient of −0.37, 95% CI −0.73 to −0.02, p value 0.041, suggesting that for each unit increase in people per bedroom there was a 0.37-point decrease in continuous SDQ score. As model assumptions were not met, caution is needed in interpretation of this finding which is not considered to represent a clinically meaningful change in child mental health.

### Effect modification by green space measures

We found no change in association between people per bedroom and SDQ category among those with differing levels of parental satisfaction with green spaces surrounding their home at the study end (table 4).

**Table 4:** Effect modification of the association between household crowding and child mental health by satisfaction with green space and ethnicity, n=3543

Variable	Increasing SDQ category, OR (CI)	P value*
People per bedroom by green space		0.168
Satisfied	0.97 (0.81 to 1.17)	
Neutral	0.63 (0.34 to 1.00)	
Dissatisfied	0.78 (0.51 to 1.19)	
People per bedroom by ethnicity		0.017
Asian	0.93 (0.77 to 1.13)	
White	0.97 (0.71 to 1.33)	
Other	0.31 (0.13 to 0.71)	

* Lrtest for effect modification

CI, confidence interval; OR, odds ratio; SDQ, strengths and difficulties questionnaire.

Sensitivity analysis showed no notable effect modification by other measures of green space including usage of parks (study end) (lrtest p 0.193), NDVI (lrtest p 0.975), distance to nearest public park or garden (lrtest p 0.208) and perceived safety of outdoor play spaces (lrtest p 0.607) (online supplemental table 5d).

### Effect modification by ethnicity

The association between people per bedroom and SDQ category was similar in the largest ethnic categories within our study: Asian and White groups (table 4). In contrast, non-White and non-Asian ethnic children appeared to have lower odds of having high SDQ scores with increasing people per bedroom (table 4). This apparent difference was not replicated in sensitivity analyses with other exposure measures including people per room (lrtest p 0.164) or square metres per person (lrtest p 0.479).

## Discussion

In this analysis of 3543 children from Bradford, there was no evidence of association between increasing household crowding at birth and worse child mental health aged 7–14 years, nor evidence for modification of the relationship by green space measures or between the main ethnic groups in the cohort.

*Several limitations* need to be considered.

The population is from one city with specific characteristics. The cohort had a higher proportion of residents of South Asian ethnicity (62%) compared with 9% in the UK overall,^33^ with under-representation of certain ethnicities including Black and non-South Asian groups. Using non-equivalised IMD categorisation for UK-wide comparison, 68% of participants lived in the most deprived quintile. The UK census 2021 estimated 4.4% of families in England to be overcrowded based on the bedroom standard.^34^ Although not directly comparable, 14% of our study participants were deemed ‘overcrowded’ using more than two people per bedroom. As a result, our findings may not be generalisable to populations with different ethnic compositions, socioeconomic profiles and housing conditions.

Only a subset (43%) of the original cohort was followed up. Mothers who experienced both household crowding and whose children had poor mental health may have been less likely to engage in follow-up than mothers experiencing one of these, leading to participation bias.

While we used several measures of household crowding, we did not have sufficient data to apply the bedroom standard used widely in UK housing policy (10), which considers the composition of the household in its calculations of occupancy levels. This would have enabled us to adjust for household composition including age and relatedness of household members. Feedback from Bradford residents indicated that large multi-generational were felt to provide support, opportunities for socialisation and a sense of family unity and was cited as a reason why families might choose to live in more crowded conditions in the context of low availability and affordability of housing. While household composition could give some indication of support, nuances in family relationships and dynamics and support from families and friends living nearby would require dedicated questions regarding the nature and perception of support.

Despite being a validated measure of child mental health, the parent completed SDQ may be subject to biases. For example, maternal mental health may influence how mothers perceive their child’s mental health. Furthermore, we only had SDQ data for one point in time (the study end). As a result, we were unable to perform longitudinal modelling of mental health trajectories over time from the date of initial exposure.

Our study may not have been sufficiently powered to pick up very small effect sizes of less than 0.05-point increase in behavioural score per unit increase in person per room, as observed previously.^9^

Sparsity may have affected subgroup analyses. For example, while we observed a positive association between increasing household crowding and lower SDQ scores among those of non-White and non-Asian ethnicity, there were small numbers of children with borderline and raised SDQ scores in this heterogeneous ethnicity category.

It is possible that residual confounding may explain the findings, due to low completeness of included variables, or unmeasured variables. For example, low completeness of variables pertaining to use of green spaces and garden access made it hard to determine the protective role of green space use. Furthermore, for those green space variables that were sufficiently complete, measurement occurred at the study end and was treated as a proxy for green space exposure at the study start when our primary exposure was measured. Additionally, despite adjusting for a range of confounders, it is possible that residual confounding exists, household crowding often co-occurs with other internal housing issues including damp and housing disrepair, which were not captured in our study. While each element alone may have small or insignificant effects, crowding—like disrepair—but could contribute to overall negative effects of household disadvantage on child well-being.^11,35^ Adverse childhood experiences, known to correlate with child mental health, may also associate with household crowding but we did not have the data to adjust for these here.

While our main analytic approach did not detect a linear relationship between household crowding and children’s mental health, a possible explanation for our null finding is that the association follows a complex, non-linear pattern. Possible non-linear patterns of association between SDQ category and differing levels of crowding were explored using household crowding measures in categorical forms, online supplemental table 5a, however these were exploratory and inconsistent between variables.

Our study has several *strengths*.

It used a longitudinal cohort permitting us to observe effects of household crowding at birth on mental health outcomes later in a child’s life and explore effects of cumulative and changing levels of crowding.

The richness of the Born in Bradford data permitted us to explore a wide range of confounders and effect modifiers. Sensitivity analyses using differing assumptions were broadly consistent with the main findings.

To our knowledge this was the first UK study exploring associations between child household crowding and child mental health in a deprived and ethnically diverse community.

We discuss *possible explanations* for our findings informed by previous studies and our discussions with the community; critical periods for exposures and outcomes; variations in social norms around living arrangements between populations; advantages of living in smaller homes with more family members; and finally, the role of subjective perceptions of crowding in affecting mental health.

Regarding critical periods for exposure, crowding was measured at birth, and mental health was measured when children in our study were on average 10 years old (range 7–14 years). Children in the Southampton Women’s survey, UK^10^ which found an increase of 0.2 people per room was associated with a 0.2 increase in SDQ score (95% CI 0.08, 0.32), were age 2 when crowding was measured and 3–4 years on collection of mental health outcomes. Another, US study,^9^ used cross-sectional and longitudinal cohorts and measured mental health outcomes between 3–17 years (~20% under 5 years). The study found small effect sizes in the cross-sectional cohort and no strong or consistent evidence of association in the longitudinal cohort.^9^ While our age-stratified analysis does not support substantial age-specific effects, small numbers in age-subgroups produced imprecise estimates (online supplemental table 5e). Younger children might be more affected by household crowding because they spend more time in the home compared with school-aged children. It is possible that early exposure to overcrowding becomes less important as children become older and are exposed to other determinants of mental health outside of the home environment.

It is possible that our findings differ from studies conducted in other populations because different populations have varying tolerances for household crowding linked to social and/or cultural norms. In the Born in Bradford cohort there were higher overall levels of crowding (median 1.00 people per room (IQR 0.75–1.25)) than Southampton Women’s study cohort (median 0.75 people per room (IQR 0.60–1.00)). The Born in Bradford cohort was different in other ways to the Southampton Women’s study cohort, for example, 31% versus 96% of white ethnicity; 61% of mothers had received education at A-level or above compared with 42%.

It is possible that crowding can lead to perceived beneficial effects on child mental health and wider family well-being. In discussion of the study findings, Bradford residents cited support and socialisation opportunities as benefits of living in larger family units, alongside saving money on accommodation. However, they also raised concerns about greater levels of stress and conflict due to lack of space to do homework, play and unwind in privacy, in line with qualitative studies.^7,36^ Families felt a ‘trade-off’ was required due to low availability and affordability of housing. It is possible that protective effects of living in larger family units in some communities can offset potential negative effects of reduced space leading to an overall neutral effect of increasing people per bedroom. This may explain why we observed signs of a possible protective effect of household crowding among non-white, non-Asian ethnic groups. It is possible there are distinct benefits to well-being from cohabiting with extended family when residents are relatively isolated from others in their ethnic group in the wider community. This finding requires caution, however, due to the sparsity and heterogeneity within this group.

The standardised, objective measures of crowding used in this study—while policy relevant—may not adequately capture the aspects of living in overcrowding that affect mental health. Discussions with residents identified a plethora of factors not captured by the Born in Bradford study that may contribute to subjective perception of household crowding such as household layout, condition, number of bathrooms, relatedness and personality of household members. Cultural and social factors including perceived ‘trade-offs’ associated with living in crowded accommodation discussed above may feed into how crowding is perceived and how it affects mental well-being. The relationship between objective and subjective crowding is not fully understood but research suggests that objective measures alone may miss nuances in the relationship between household crowding and mental health in adults.^36,37^

Our study has *implications for future research*. Perceived family support and household composition are required to account for potential confounding by family support. Subjective measures of perceived overcrowding alongside objective measures are required to determine their relationship with one another and relevance to mental health outcomes. To understand critical periods for exposure to crowding and timing of effects on mental health, future studies would ideally measure exposure and outcome at frequent intervals over the course of childhood. Comparison of perceived norms and stigma related to household crowding between different areas of the UK and beyond would clarify the extent to which these factors determine the effects of household crowding on child mental health. Finally, interaction between indoor and outdoor spaces in relation to child mental well-being remains under explored.

*Overall*, the relationship between household crowding and child mental health is complex. In this study, of an ethnically diverse population where crowding was relatively prevalent, no relationship was found. Household composition, age, housing-related features like layout and state of disrepair, family and community support, likely play important roles.

## Supplementary material

10.1136/bmjph-2026-005483 online supplemental file 1

## Data Availability

Data are available upon reasonable request.
