# Supplementary material for: Association between household crowding at birth and mental health outcomes in school-aged children: retrospective analysis of data from the Born in Bradford cohort, UK
Source: BMJ Public Health. 2026 Oct 29;4(4):e005483. doi: 10.1136/bmjph-2026-005483 (PMC13723880; doi:10.1136/bmjph-2026-005483)
Supplement: Supplementary file 1 [file bmjph-4-4-s001.docx]

**Appendix**

**Figure 1: Directed Acyclic Graph (DAG) depicting assumptions about covariates in the association between household crowding and child mental health:**

**Table 1: DAG variables and assumed relationships**

| **Variable** | **Classification** | **Type** | **Timing** | **Assumed relationship with household crowding (reference)** | **Assumed relationship with** Child mental health **(reference)** |
| --- | --- | --- | --- | --- | --- |
| Household crowding (People per bedroom, PPB) | Exposure | Continuous | Start | n/a | PPB -> SDQ (9, 10) |
| Child mental health (SDQ score) | Outcome | Ordinal | End | PPB -> SDQ (9, 10) | n/a |
| Age (months) | Confounder | Continuous | End | * | Age -> SDQ (11) |
| Gender | Confounder | Binary | Start | * | Gender -> SDQ (20) |
| Ethnicity | Confounder, effect modifier | Categorical | Start | Ethnicity -> PPB (3) | Ethnicity -> SDQ (20) |
| Self-reported financial status (FS) | Confounder | Binary | Start | FS -> PPB (4) | FS -> SDQ (20) |
| Index of Multiple Deprivation (IMD) | Confounder | Categorical | Start | IMD -> PPB (4) | IMD -> SDQ (20) |
| Marital/cohabitation status (MS) | Confounder | Categorical | Start | MS -> PPB (34) | MS -> SDQ (20) |
| Maternal education (ME) | Confounder | Ordinal | Start | ME -> PPB (9, 10) | ME -> SDQ (20) |
| Maternal age (years) (Mat age) | Confounder | Continuous | Start | Mat age -> PPB (1, 3, 33) | Mat age -> SDQ (39) |
| Tenure | Confounder | Categorical | Start | Tenure -> PPB (2, 3, 25) | Tenure -> SDQ (28, 35) |
| Satisfaction with green spaces (SGS)* | Effect modifier | Ordinal | End | * | SGS -> SDQ (14) |
| Neighbourhood quality (NQ)* | - | NA | NA | * | NQ -> SDQ (14) |
| Maternal mental health (GHQ score) | Mediator | Continuous | Start | PPB -> GHQ (2, 26) | GHQ -> SDQ (38) |
| Number of moves | Mediator | Discrete (count) | End | PPB -> moves (25) | Moves -> SDQ (25, 28, 40) |
| Home disrepair | Mediator | NA | NA | PPB -> disrepair (2, 25) | Disrepair -> SDQ (5) |

PPB = People per bedroom

SDQ = Strengths and difficulties questionnaire

GHQ = General health questionnaire

NA = Not available due to the variable not being measured

*No direct association with people per bedroom assumed

**Figure 2: Distribution of household crowding using people per bedroom**

**Table 2: Characteristics of participants with respect to additional variables used in sensitivity analysis:**

| Variable | Variable summary: Number (%) unless otherwise stated | Number of missing observations from a total of 3913 |
| --- | --- | --- |
| Alternative household crowding measures | | |
| People per bedroom (binary, overcrowding)  </= 2 people per bedroom  >2 people per bedroom | 3285 (85)  577 (15) | 51 |
| People per bedroom (tertiles, defined by data)  </=1.3 people per bedroom  >1.3 to <1.8 people per bedroom  >/= 1.8 people per bedroom | 922 (24)  1731 (45)  1209 (31) | 51 |
| People per bedroom study end (median, IQR) | 1.50 (1.3-1.8) | 222 |
| Change in people per bedroom (start to end)  Decrease  No change  Increase | 1171 (32)  753 (21)  1731 (47) | 258 |
| Cumulative people per bedroom (start and end)  Never >2 people per bedroom  >2 people per bedroom at start or end  Always >2 people per bedroom | 2913 (80)  641 (17)  101 (3) | 258 |
| People per room (median, IQR) | 1.00 (0.8-1.3) | 1029 |
| Square metres per person (median, IQR) | 20.20 (15.8-26.0) | 1040 |
| Additional covariates | | |
| Maternal GHQ-28 score study start (mean, SD) | 23.56 (10.6) | 652 |
| Number of moves  No moves  One move  2-3 moves  >3 moves | 1776 (45)  1141 (29)  775 (20)  221 (6) | 0 |
| Alternative green space measures | | |
| Usage of parks  Rarely  Some weekdays or some weekends  Some weekdays and some weekends  Most days | 753 (29)  969 (38)  651 (25)  220 (8) | 1320 |
| NDVI (mean, SD) | 0.22 (0.06) | 150 |
| Perceived presence of safe outdoor space  Yes  No | 3584 (95)  198 (5) | 131 |
| Distance to nearest park, metres (mean, SD) | 893.10 (553.9) | 150 |

SD, standard deviation

IQR, interquartile range

GHQ, General Health Questionnaire 28

NDVI, normalised difference vegetation index

**Table 3: Features of those who participated in the baseline survey but did not complete follow up compared to those who participated in both the baseline survey and follow up**

Note, all available data has been used, the total number of observations vary according to the completeness of the given variable.

| Variable | Participants who participated in the baseline survey but were not followed up  N=6382  Number (%) unless otherwise stated | Number of missing observations | Participants who were recruited to and participated in follow up  N= 3913  Number (%) unless otherwise stated | Number of missing observations | P value for difference between groups* |
| --- | --- | --- | --- | --- | --- |
| Gender  Male  Female | 3289 (52)  3093 (48) | 0 | 2002 (51)  1911 (49) | 0 | 0.713 |
| Ethnicity  White  Asian  Other | 3013 (47)  2770 (43)  597 (9) | 2 | 1229 (31)  2437 (62)  247 (6) | 0 | <0.001 |
| Self-reported financial status  Not struggling  Struggling | 4307 (68)  2028 (32) | 47 | 2696 (69)  1198 (31) | 19 | 0.187 |
| IMD  4/5 (least deprived)  3  2  1 (most deprived) | 1099 (17)  1210 (19)  1730 (28)  2236 (36) | 107 | 579 (15)  730 (19)  1074 (28)  1499 (39) | 31 | 0.001 |
| People per bedroom  Mean (SD) | 1.63 (0.59) | 56 | 1.65 (0.58) | 51 | 0.094 |

IMD, index of multiple deprivation equivalised for Bradford

SD, standard deviation

N, number

*Pearson chi squared test or two-sided t-test

**Table 4:** **Features of those who participated in both baseline and follow-up surveys comparing those with complete and in-complete data**

| Variable | Study end complete data  Number (%) or  Mean (SD) | Number of observations | Study end incomplete data*  Number (%) or  Mean (SD) | Number of observations | P (for difference between groups)** |
| --- | --- | --- | --- | --- | --- |
| Gender  Male  Female | 1799 (51)  1744 (49) | 3543 | 203 (55)  167 (45) | 370 | 0.134 |
| Age (months) | 118.21 (12) | 3543 | 120.51 (12) | 370 | <0.001 |
| Ethnicity  Asian  White  Other | 2207 (62)  1128 (32)  208(6) | 3543 | 230 (62)  101 (27)  39 (11) | 370 | 0.001 |
| Self-reported financial status  Not struggling  Struggling | 2446 (69)  1097 (31) | 3543 | 250 (71)  101 (29) | 351 | 0.397 |
| IMD  1 (most deprived)  2  3  4 (least deprived) | 1348 (38)  986 (28)  674 (19)  535 (15) | 3543 | 151 (45)  88 (26)  56 (17)  44 (13) | 339 | 0.125 |
| People per bedroom | 1.65 (0.58) | 3543 | 1.67 (0.61) | 319 | 0.404 |
| SDQ score  Normal (0-13 points)  Borderline (14-16 points)  Raised (17-40 points) | 2873 (81)  265 (8)  405 (11) | 3543 | 256 (79)  25 (8)  42 (13) | 323 | 0.782 |

*Note, for those with incomplete data, the total number of observations vary according to the completeness of the given variable.

**Chi-squared test for categorical variables and t-test for continuous variables

SD, standard deviation

IMD, index of multiple deprivation equivalised for Bradford

SDQ, strengths and difficulties questionnaire

**Tables 5a-e:** **Sensitivity analyses for the association between household crowding and child mental health using alternative measures of crowding and additional or alternative confounders and effect modifiers**

Note, all models in this table include the covariates included in our final, fully adjusted logistic regression model: Age, sex, ethnicity, IMD, self-rated financial status, maternal education, maternal cohabitation status, tenure, maternal age, satisfaction with green spaces.

In the case of comparison of green space measures, satisfaction with green spaces is replaced with alternative green space measures.

To ensure comparability only participants with complete data in all measures of crowding were used for each individual analysis.

a) **Alternative groupings of household crowding measures into binary and tertile forms, n=2644**

| Variable | Increasing SDQ category, OR (CI) | P value* |
| --- | --- | --- |
| People per bedroom (binary)  <=2.00  >2.00 | 1.00  0.82 (0.62-1.10) | 0.188 |
| People per bedroom (tertiles, defined by data)  <=1.3  >1.3 and <1.8  >/= 1.8 | 1.00  1.22 (0.93-1.60)  0.96 (0.71-1.29) | 0.148  0.780 |
| People per room (binary)  <=1.00  >1.00 | 1.00  1.07 (0.86-1.33) | 0.558 |
| People per room (tertiles)  <=0.8  >0.8 and <1.1  >/=1.1 | 1.00  1.35 (1.04-1.76)  1.29 (0.97-1.71) | 0.024  0.077 |
| Square metres per person (binary)  >/=15.3  <15.3 | 1.00  1.07 (0.83-1.36) | 0.605 |
| Square metres per person (tertiles)  >/=23.8  <23.8 and >17.3  <=17.3 | 1.00  1.22 (0.95-1.57)  1.29 (0.99-1.67) | 0.123  0.055 |

*Two-sided Wald z-test

SDQ, strengths and difficulties questionnaire

OR, odds ratio

b) **Alternative time points for measuring people per bedroom including study end, change over time and cumulative exposure, n=3446:**

| Variable | Increasing SDQ category, OR (CI) | P value* |
| --- | --- | --- |
| People per bedroom (measured at study end) | 0.91 (0.74-1.12) | 0.357 |
| Change in people per bedroom (study start to study end)  Decrease  No change  Increase | 1.00  1.29 (1.01-1.65)  1.00 (0.82-1.65) | 0.045  0.978 |
| Cumulative overcrowding study start to end  <=2 people per bedroom at both start nor end  >2 people per bedroom at either start or end  >2 people per bedroom at start and end | 1.00  0.95 (0.74-1.20)  0.678 (0.52-1.27) | 0.641  0.220 |

* People per bedroom at the study start was moderately correlated with people per bedroom at the study end (Pearson’s correlation coefficient 0.31).

SDQ, strengths and difficulties questionnaire

OR, odds ratio

c) **Use of additional covariates: maternal mental health and number of moves, n=2972:**

| Variable | Increasing SDQ category, OR (CI) | P value* |
| --- | --- | --- |
| People per bedroom, original model | 0.85 (0.71-1.02) | 0.085 |
| People per bedroom + maternal mental health included as covariate | 0.84 (0.70-1.01) | 0.065 |
| People per bedroom + number of moves included as covariate | 0.85 (0.70-1.02) | 0.084 |
| People per bedroom + maternal mental health + number of moves, included as covariates | 0.84 (0.69-1.01) | 0.068 |

*Two-sided Wald z-test

SDQ, strengths and difficulties questionnaire

OR, odds ratio

d) **Use of alternative measures of green space: park usage, NDVI, perceived outdoor safety, distance to nearest park, n=2290:**

| Variable | Increasing SDQ category, OR (CI) | P value* |
| --- | --- | --- |
| People per bedroom by usage of parks  Rarely  Some weekdays or some weekends  Some weekdays and some weekends  Most days | 0.81 (0.56-1.19)  0.78 (0.54-1.13)  0.82 (0.55-1.23)  1.67 (0.91-3.10) | 0.193 |
| People per bedroom by NDVI | 0.87 (0.70-1.08) | 0.975 |
| People per bedroom by perceived presence of safe outdoor space  Yes  No | 0.87 (0.70-1.09)  0.70 (0.31-1.60) | 0.608 |
| People per bedroom by distance to nearest park | 0.87 (0.70-1.08) | 0.208 |

*Lrtest for effect modification

SDQ, strengths and difficulties questionnaire

OR, odds ratio

NDVI, normalised difference vegetation index

**e) Stratified analysis by age group categories**

| Variable | Increasing SDQ category, OR (CI) | P value* | Number of observations |
| --- | --- | --- | --- |
| Age  Under 9 years  9 to 11 years  Over 11 years | 0.93 (0.65-1.33)  0.85 (0.69-1.04)  1.18 (0.63-2.24) | 0.693  0.112  0.602 | 673  2480  293 |

*Two sided Wald z-test

SDQ, strengths and difficulties questionnaire

OR, odds ratio
